# Design, implementation, and evaluation of a PRECEDE-PROCEED model-based intervention for oral and dental health among primary school students of Rafsanjan city: a mixed method study

**DOI:** 10.1186/s12889-021-11585-z

**Published:** 2021-09-03

**Authors:** Mohtasham Ghaffari, Sakineh Rakhshanderou, Mohammad Asadpour, Mostafa Nasirzadeh, Leili Mazar

**Affiliations:** 1 Department of Public Health, School of Public Health and Safety, Shahid Beheshti University of Medical Sciences, Tehran, Iran; 2 Department of Health Education and Health Promotion, School of Health, Rafsanjan University of Medical Sciences, Rafsanjan, Iran; 3 Department of Health Education and Health Promotion, School of Health, Occupational Environment Research Center, Rafsanjan University of Medical Sciences, Rafsanjan, Iran; 4 Department of Health Education and Health Promotion, School of Health, Student Research Committee, Rafsanjan University of Medical Sciences, Rafsanjan, Iran

**Keywords:** Oral and dental health, Brushing, PRECEDE-PROCEED model, Intervention, Primary schools’ students

## Abstract

**Background:**

Oral and dental health has a significant impact on public health as well as the quality of life among individuals and families. This study aims to design, implement, and evaluate an intervention based on the PRECEDE-PROCEED model for oral and dental health among primary school students in Rafsanjan city, Southern Iran.

**Methods:**

According to the nature of the model and with the focus group discussion and interview methods, in phases 1 to 4 (PRECEDE), predisposing, enabling, and reinforcing factors related to oral health were identified. The training program was designed and consisted of six sessions for students (250 students) with a brushing session, three sessions for parents, and two sessions for teachers. Process evaluation and the effect of the program on behavioral and factors affecting oral health were evaluated (PROCEED).

**Results:**

In the qualitative section, seven predisposing factors, five enabling factors, and two reinforcing factors were identified. A training program based on the PRECEDE-PROCEED model was found to be effective in increasing the mean scores of the above constructs and the students’ brushing behavior (*P* < 0.001).

**Conclusion:**

Based on the model planning phases, the factors affecting the brushing behavior of children aged 6–12 years were identified. The educational program has had a significant effect on improving the scores of predisposing, enabling and reinforcing factors and children’s brushing behavior.

**Supplementary Information:**

The online version contains supplementary material available at 10.1186/s12889-021-11585-z.

## Background

Oral and dental health has a significant impact on public health as well as the quality of life among individuals and their family [1, 2]. The World Health Organization (WHO) defines oral and dental health as “ a person’s status being free from any acute and chronic diseases in the mouth, teeth, face, and throat, which limit their capacity for nutrition, smile, speech, as well as mental and social health” [3].

About 99% of people develop dental caries throughout their lives, and around 37% of their teeth are destroyed due to caries [4]. Oral and dental diseases have been the most common diseases in humans, especially in children [5], with one out of every four preschool children in the United States suffering from tooth decay [6]. The latest results from oral and dental examinations of children aged 3 to 6 show that Iranian children pass their 3-year-old age with about 2 decayed primary teeth, and their 6-year-old age with 5 decayed primary teeth [4].

Various barriers have been reported to oral and dental health among children, including high costs, low self-efficacy, fear, false beliefs about primary teeth repair, lack of cooperation and time for healthcare issues, lack of knowledge, and having a poor attitude towards oral and dental health [7–9]. Improper nutritional behavior in children, such as high sugar consumption, lack of dairy consumption, and the non-internalization of habits, such as being reluctant to brush and use dental floss lead to a high incidence of caries [10, 11]. The household’s economic status [5] as well as the family’s high education level promote children’s oral and dental health [12, 13].

Children’s oral and dental health is influenced by parental behavior; in addition, the interaction between family members has a positive impact on children’s perception of oral and dental health [14]. Children’s educational performance is highly influenced by their oral and dental health. Thus, children with poor oral and dental health are 12 times more likely than children with good oral and dental health to have restricted days, such as absenteeism [15].

Education is one of the strategies for the prevention of oral and dental diseases; hence, to achieve encouraging results, education must be principled and based on behavioral change patterns and theories [16]. The PRECEDE-PROCEED model is a planning, participatory, and community-based model widely used in planning and changing behavior and its purpose is to attract people’s partnership, to utilize the specialist’s view on planning, and to have a comprehensive look at the behavioral and non-behavioral determinants. This model was introduced by Green et al. and has eight phases in two parts [17], including four planning phases, one implementation phase, and three evaluation phases. The PRECEDE part of this model stands for predisposing, reinforcing, and enabling constructs in terms of educational diagnosis and evaluation, which has an educational aspect. The PROCEED section focuses on health promotion aspects, including policies, regulations, and organizations in environmental and educational development. This model examines the causal relationship in the analysis of a health problem, but it does not measure the relationship between cause and effect and provides a clear framework for developing a behavior change plan [18].

As WHO approved the attempts to increase building capacities for education, influencing health-related behaviors namely knowledge, beliefs, skills, attitudes, values, support, and making healthy decisions, school can be one of the most suitable places for health promotion [19].

Due to the failure to achieve WHO’s goals for children’s oral and dental health, this study aims to design, implement, and evaluate an intervention based on the PRECEDE-PROCEED model for oral and dental health among primary school students in Rafsanjan city.

## Methods

### Study setting and participants

The present study is an exploratory sequential study consisting of two qualitative and quantitative stages conducted in 2016–2017 over a 15-month period.

In the qualitative stage, a sample of 39 volunteers, including dentists, health education and promotion professionals, parents and teachers, as well as school health educators participated through purposeful sampling with maximum variation and a semi-structured interview was conducted to determine predisposing, enabling, and reinforcing factors related to the priority identified at the third phase, data were analyzed by content analysis.

In the quantitative stage, a total of 250 students (*n* = 122 in the intervention group and *n* = 128 in the control group) studying in the primary school second grade in Rafsanjan city, were enrolled in the study. The sample size was calculated using the formula (*n* = (*Z*_1_ + *Z*_2_)^2^× (2*S*^2^) /d^2^) considering a 10% chance of attrition. The sampling method was multi-stage cluster sampling. After coordinating to the authorities and preparing a list of schools by the executor of the project (M.N), in the first step, two girls’ and boys’ schools (four schools in total) considered randomly from 23 state schools (12 girls’ schools and 11 boys’ schools) in Rafsanjan. In the second step, based on the determined sample size and the list of students in each school, one class in each grade (fourth, fifth and sixth) was randomly selected.

### Study procedure and data collection

Considering the planning essence of this model the following steps are taken corresponding to the phase number one to four:

In the social assessment (phase 1), the impact of oral and dental health on the quality of life in children and their families was assessed in different aspects based on a focus group discussion with 9 stakeholders (mothers with children aged 6–12 and dentists) and the study of 13 theses, 26 Persian articles, 54 English articles, 12 books, other scientific resources, and It was found out that poor oral and dental health affects people’s physical, mental, social, and economic quality of life (Table 1).

**Table 1:** Explaining the Importance of Oral Health, Inappropriate oral health, and its impact on quality of life from the perspective of literature review

Dimensions of quality of life	Example	References
Physical dimension	The incidence of diseases, halitosis, speech and taste impairment, reduced growth, the risk of malnutrition, sleep and smile disturbance, impairment in learning, failure to do daily homework, disorder in the game	[20–23]
Psychological dimension	Reduction of self-esteem and self-confidence, incidence of anxiety, stress, depression, feeling restless and chaotic, dissatisfaction with mental imagery and general appearance, lack of sense of well-being and emotional health, humiliation feeling, shame and embarrassment	[24–26]
Social dimension	Disruption of communication and social interactions, lack of social participation (like recreational programs), social rejection and labeling, absence from school and work, disruptions in career and academic performance, impact on the quality of family life	[27–29]
Economic dimension	Impact on the economic situation of the family, impose economic costs	[30, 31]

In the epidemiological, behavioral, and environmental assessment (phase 2), the major index related to the quality of life, in terms of oral and dental health, was the number of decayed, missing (extracted), and filled teeth (DMFT), and the most important of which was dental caries. Next, behavioral and non-behavioral factors were identified (Table 2) based on a focus group discussion with 9 stakeholders (mothers with children aged 6–12 and dentists), and the review of 9 theses, 36 Persian articles, 39 English articles, 8 books, other scientific resources, such as the World Health Organization. In addition, the major influential behavioral factor was determined to be brushing behavior based on the opinions of a 14 health education professionals and dentists on the basis of decision-making matrix, with two criteria of importance and variability.

**Table 2:** Behavioral and non-behavioral factors affecting tooth decay based on literature review and scientific resources

Behavioral factors	Non-behavioral factors
Do not do fluoride therapy	Person and tooth resistance (genetic factors, congenital or acquired)
Use of fluoride-free toothpaste	Fluoride in drinking water
No use of dental floss	Age and gender
Do not brush	Economic status and social class
Over-consumption of sugar, sweets, and carbohydrates	Dimensions of the family
Lack of regular referral (six months) to the dentist to receive preventive services	Parent’s job and education
Overeating and weight gain	blood type
Reduce milk and dairy consumption	Level of salivation and concentration
Eating snacks and unhealthy snack foods -chewing Gum, Chips, Puffs, Lavashes and Salt and Nutritional Snacks- junk food	Chronic and systemic diseases
	Residence
	Consistency of food
	Health literacy

In the educational and ecological assessment (phase 3), predisposing, enabling, and reinforcing factors associated with brushing behavior were identified based on a focus group discussion with 9 stakeholders (mothers with children aged 6–12 and dentists) and the study of 7 theses, 26 Persian articles, 21 English articles, books, scientific websites and documentations, and next, a qualitative study was designed aimed at better explaining the data, and semi-structured interviews were conducted with 39 stakeholders, including dentists, health education and promotion professionals, parents, teachers, and school health educators. Accordingly, the major factors affecting tooth-brushing behavior were identified, and after adjusting them to the findings of the first part of this phase, the major predisposing, enabling, and reinforcing factors associated with brushing behavior were identified.

All the executive activities of the above steps were performed by the project executor (M.N) and the actions were approved by the other members of the research team.

After adjusting the findings of the qualitative stage to those of the quantitative one, the questionnaire was formulated, face and content validity was assessed by 10 experts in health education and health promotion and pediatric dentistry, and reliability was assessed by a test-retest estimate in 57 students at a 14-day interval, and the final tool was designed (Table 3). The questionnaire is available in supplementary section (Additional file 1).

**Table 3:** The characteristics of the tools developed in three areas predisposing, enabling, and reinforcing factors

Factor	Structure	Number of questions	Response scale	How to respond	The scope of questions	CVR	CVI	Cronbach’s alpha	Internal correlation coefficient
*Demographic characteristics		11	–	Self-Reporting	–	–	–	–	
	**Knowledge**	24	multiple choice	Self-Reporting	0–24	0.95	0.96	–	0.84
**Predisposing Factors**	Attitude	7	Three-part Likert	Self-Reporting	7–21	0.94	0.97	0.72	
	Perceived susceptibility	3	Three-part Likert	Self-Reporting	3–9	1	0.93	0.68	
	Perceived severity	10	Three-part Likert	Self-Reporting	10–30	0.96	0.96	0.90	
	Perceived benefits	11	Three-part Likert	Self-Reporting	11–33	1	0.99	0.78	
	Subjective norms	3	Three-part Likert	Self-Reporting	3–9	1	1	0.73	
	Observational learning	3	Three-part Likert	Self-Reporting	3–9	1	1	0.85	
	The motivation to comply	3	Three-part Likert	Self-Reporting	3–9	1	1	0.88	
**Enabling Factors**	Perceived barriers	6	Three-part Likert	Self-Reporting	6–18	1	1	0.80	
	Self-efficacy	5	Yes - to some extent - no	Self-Reporting	5–15	1	1	0.78	
	Perceived behavioral control	6	Yes - to some extent - no	Self-Reporting	6–18	0.96	0.98	0.79	
	Behavioral preferences	5	Three-part Likert	Self-Reporting	–	1	1	–	
	Brushing skill	12	Yes No	Checklist- Observation	0–12	–	–	0.82	
**Reinforcing Factors**	Social reinforcement	5	Three-part Likert	Self-Reporting	5–15	1	1	0.85	
	Social support	4	Three-part Likert	Self-Reporting	4–12	1	1	0.76	
	**Behavior**	21	Yes- No	**check list-	0–21	–	–	–	
	**Behavioral intention**	4	Yes- No	Self-Reporting	0–4	0.95	1	0.82	

*Demographic variables included age, sex, education, and parents’ occupation, family income and oral health status

** Parents completed the checklist of behavior

In the administrative & policy assessment and intervention alignment (phase 4), project executor (M.N), held a session at the office of the head of Rafsanjan Administration of Education, and the program was explained to the principals of the selected schools. Then, a coordination session was held separately at each school, all resources and facilities were reviewed, and pre-test was done. The results of the pre-test were analyzed, an intervention program was formulated, a coordination session with school administrators was re-arranged, and the method of implementing the intervention program was explained.

In the process evaluation phase, we evaluated the implemented program according to what we had designed, the designed program proceeded regularly, only the interviews required more time. An intervention program was run based on the findings from previous steps among students (6 training sessions and one session for brushing) aimed at promoting students’ knowledge, attitude, and practice in terms of brushing by the executor of the project (M.N). The program included lectures, questions, and answers, the explaining of related experiences, practical demonstration, and role playing in learning areas and educational goals, the use of educational media, such as short videos, posters, and educational folders. Three sessions for parents and two sessions for teachers and school health educators were held aimed at enhancing their collaboration and gaining their support for sustaining students’ behavior. Then, impact assessments were carried out, based on the designed method, among the students 3 months after the intervention. In addition, leaflets, CDs, educational folders, toothbrushes, and toothpastes were distributed among the students as gifts.

In the brushing session, according to the correct brushing method at the last session using practical demonstration, the role-playing method, and the use of moulage, the school water supply was used, and brushing was performed in groups of 5 to 7 individuals, with the brushing behavior of each student evaluated and necessary trainings provided. The purpose of this session was to remove some barriers, such as embarrassment and low self-efficacy in some students. In this session, the school’s health educator brushed with the students, thereby encouraging them to grow enthusiasm.

In the beginning of the study (phase 1) until the completion of the intervention program (the implementation phase), all activities were planned in the Gantt chart. This program was scheduled for a 12-month period, and process evaluation was carried out and reported for each activity. To do so, activities carried out at each phase were reviewed by the type of activity, its scheduling, and its implementation method, with necessary corrections made. The important point in the evaluation was the increase in the time assigned to the interviews, which were planned over 2 months, but they took 3 months due to the sampling problems. In the end, it took 12 months for this study to complete the intervention and 3 months to evaluate the effect.

### Statistical analyses

Data were analyzed by SPSS version 18.0 using Mann-Whitney, Wilcoxon, and chi-square tests at the significance level of 0.05.

## Results

Based on the essence of model planning and according to phases one to 5 of the model, as described in the materials and methods section, an intervention program was designed and implemented; the results of the program are as follows:

According to the impact assessment, the mean age of the intervention group and that of the control group was 10.77 ± 1.01 and 10.98 ± 0.88, respectively. About 49 and 47% of the students in the intervention and control groups were girls, respectively. The results of the independent t-test and the chi-square test showed no significant difference in the variables of age, gender, parental education, parental occupation, family income, as well as oral and dental health status between the two groups (*P* > 0.05). After implementing the intervention program, a significant difference was observed (*P* < 0.001) in the mean score and standard deviation of knowledge, attitude, perceived susceptibility, perceived severity, perceived benefits, perceived barriers, self-efficacy, perceived behavioral control, subjective norms, motivation to comply, observational learning, brushing skills, social support and reinforcement, as well as the behavioral intention of brushing in the intervention group members. In addition, the results indicated the impact of the intervention program on all predisposing, enabling, and reinforcing factors as well as proper brushing behavior (Tables 4 and 5).

**Table 4:** Comparison of the mean score of predisposing, enabling, and reinforcing factors before and 3 months after the intervention in two groups

Factor	Structure	Group	Before of intervention	After of intervention	*P*-Value*
			M ± SD	M ± SD	
Predisposing Factors	Knowledge (0–24)	Intervention	10.48 ± 3.37	18.93 ± 2.77	< 0.001
		Control	10.40 ± 2.98	11.66 ± 3.54	< 0.001
		*P*-Value**	0.091	< 0.001	
	Attitude (7–21)	Intervention	18.70 ± 2.00	19.82 ± 1.99	< 0.001
		Control	18.80 ± 2.01	19.01 ± 1.81	0.14
		*P*-Value**	0.93	< 0.001	
	Perceived Susceptibility (3–9)	Intervention	7.41 ± 1.45	7.87 ± 1.40	< 0.001
		Control	7.45 ± 1.49	7.81 ± 1.27	0.005
		*P*-Value**	0.74	0.04	
	Perceived Severity (10–30)	Intervention	24.11 ± 3.89	28.36 ± 3.39	< 0.001
		Control	23.00 ± 4.22	23.44 ± 4.05	0.30
		*P*-Value**	0.05	< 0.001	
	Perceived Benefit (11–33)	Intervention	29.08 ± 3.35	31.95 ± 1.96	< 0.001
		Control	29.51 ± 3.24	29.41 ± 3.21	0.51
		*P*-Value**	0.26	< 0.001	
	Subjective Norms (3–9)	Intervention	8.26 ± 1.07	8.65 ± 0.83	< 0.001
		Control	8.22 ± 1.19	8.19 ± 1.20	0.30
		*P*-Value**	0.63	< 0.001	
	Motivation to Comply (3–9)	Intervention	7.71 ± 1.45	8.44 ± 1.13	< 0.001
		Control	7.46 ± 1.53	7.29 ± 1.62	0.23
		*P*-Value**	0.11	< 0.001	
	Observational Learning (3–9)	Intervention	7.13 ± 1.89	8.07 ± 1.63	< 0.001
		Control	7.25 ± 1.57	6.72 ± 1.86	0.007
		*P*-Value**	0.94	< 0.001	
Enabling Factors	Perceived Barrier (6–18)	Intervention	8.61 ± 2.29	7.45 ± 2.33	< 0.001
		Control	8.20 ± 1.94	8.30 ± 2.82	0.81
		*P*-Value**	0.25	< 0.001	
	Self-Efficacy (5–15)	Intervention	13.29 ± 2.29	14.36 ± 1.23	< 0.001
		Control	13.18 ± 2.22	13.17 ± 2.09	0.74
		*P*-Value**	0.49	< 0.001	
	Perceived Behavioral Control (6–18)	Intervention	14.14 ± 2.76	16.37 ± 2.16	< 0.001
		Control	14.35 ± 2.83	14.42 ± 2.93	0.92
		*P*-Value**	0.49	< 0.001	
	Tooth Brushing Skill (0–12)	Intervention	5.72 ± 2.27	10.72 ± 1.75	< 0.001
		Control	6.44 ± 2.77	5.59 ± 2.15	0.01
		*P*-Value**	0.01	< 0.001	
		**Ancova**	–	< 0.001	
Reinforcing Factors	Social Support (4–12)	Intervention	9.99 ± 2.12	11.00 ± 1.61	< 0.001
		Control	9.54 ± 2.06	9.44 ± 2.35	0.72
		*P*-Value**	0.02	< 0.001	
		**Ancova**	–	< 0.001	
	Social Reinforcement (5–15)	Intervention	12.15 ± 2.76	13.88 ± 1.85	< 0.001
		Control	12.14 ± 2.47	11.75 ± 2.92	0.14
		*P*-Value**	0.07	< 0.001	
Behavior	Intention Behavior (0–4)	Intervention	3.19 ± 1.27	3.73 ± 0.68	< 0.001
		Control	3.74 ± 0.67	3.33 ± 1.10	0.62
		*P*-Value**	0.02	< 0.001	
		**Ancova**	–	< 0.001	

*Mann Whitney U test

**Wilcoxon test

**Table 5:** Comparison of frequency distribution of brushing behavior of students before and after intervention in two groups

Daily toothbrush behavior	Group	Before intervention		After intervention		*P*-Value
		Number	Percentage	Number	Percentage	
Less than once	Intervention	27	22.1	1	0.82	< 0.001
	Control	16	12.5	14	11.02	0.09
	***P*-Value**	0.03		< 0.001		
One to two times	Intervention	16	13.1	3	2.4	< 0.001
	Control	30	23.4	27	21.2	0.08
	***P*-Value**	0,04		< 0.001		
Twice and more	Intervention	79	64.7	117	96.6	< 0.001
	Control	0.03	64	86	67.6	0.08
	***P*-Value**	< 0.001		< 0.001		

## Discussion

Based on the phases of the model, at the phase one, two, and three, using group discussion with mothers and professionals, as well as literature review, scholarly resources and dissertations, the importance of oral and dental health was explained, and the major index influencing the quality of life associated with oral and dental health was determined. In addition, the major behavioral factor influencing that index was identified. Next, the predisposing, enabling, and reinforcing factors associated with that factor were identified.

Similar to other studies, DMFT was the major index of the quality of life affecting oral and dental health [32–34]. At the beginning of the study, 35.2% of the students brushed their teeth less than twice a day, and 64.7% of them brushed their teeth at least twice a day. In the study of Goodarzi et al., 44.7% of students brushed their teeth less than twice a day, and in the study of John et al., 31.5% of students brushed their teeth at least twice a day [35, 36]. After the educational intervention, 96.6% of the students brushed their teeth at least twice a day, indicating an improvement in their brushing performance through education.

### Predisposing factors

The major predisposing factors of brushing behavior included demographic characteristics, knowledge, attitude, perceived susceptibility, perceived severity, perceived benefits, subjective norms, observational learning, and motivation to comply.

Knowledge, as the first step in the behavior change process, could play a key role in this process. The major weaknesses of the students in terms of awareness were the lack of knowledge of negative consequences (lack of awareness of psychological, psychological, and social consequences, such as decreased self-esteem, embarrassment, and losing friends), not meeting oral and dental health requirements, and the presence of symptoms of dental caries. Among the students’ wrong attitudes, one could refer to the lack of belief in the brushing method and benefits of toothpastes. Holding a group discussion, answering questions about the complications and symptoms of dental caries as well as improper oral and dental health, and also extracting and correcting some of the wrong attitude and beliefs led to an increase in the mean score of these constructs in the intervention group. In the study of Babaee et al. on students aged 12–14 and in that of Sanadhya et al. on students aged 12–15, oral and dental health education improved the mean score of students’ knowledge [37, 38]. In the study of Shirzad et al. an educational intervention could change the attitude of mothers and teachers towards students’ brushing [39].

In the present paper, by informing the students on the positive consequences of proper oral and dental health behavior as well as the negative consequences in different aspects of the quality of life, their perceived susceptibility and perceived severity improved. Peyman and Pourhaji in their study, provided an educational program based on the Health Belief Model on oral and dental health behavior, which significantly increased the scores of perceived susceptibility, perceived severity, and perceived benefits in primary school students [40]. Subjective norms are the amount of social pressure perceived by an individual to perform a behavior; in other words, it is the reflection of the social influence on the individual, along with motivation to comply, which can cause to behavioral intention [18]. After the intervention program, the difference between the mean score of subjective norms and that of motivation to comply was significant between the two groups, indicating that the intervention program increased the mean score of these two constructs. In the study by Naseri-Salahshour et al. an oral and dental health promotion program aimed at preventing early dental caries in schoolchildren led to a significant increase in subjective norms after educational intervention [41].

### Enabling factors

The major enabling factors associated with brushing behavior included perceived barriers, self-efficacy, perceived behavioral control, and the ability to brush or brushing skills.

Gum bleeding and numbness were the major perceived barriers in the present study. The mean score of perceived barriers decreased significantly by a group discussion, questions and answers, as well as the explaining of the importance of oral and dental health and its effects on general health. In the study of Hajimiri et al. oral and dental health education in mothers of children aged 3–6 in terms of tooth decay, based on the Health Belief Model in Zanjan city, significantly reduced the perceived barriers score [42]. Such a statistically significant difference was also observed in the study of Shamsi et al. on pregnant women in Arak [43].

The concept of self-efficacy in the present study was students’ confidence in the ability to correct brushing behavior after each meal, between meals, and even after eating sweets. The mean score of self-efficacy after the intervention was 95.73%, which was significantly higher than that before the intervention. Consistent with the present study, in the study of Ghorbaniet al. the mean score of the self-efficacy of oral and dental health-promoting behavior increased significantly after an educational intervention [44]. According to the results of some studies, poor self-efficacy among students regarding oral and dental health care behavior is one of the main causes of tooth decay and loss [45, 46]. In another study, an increase in self-efficacy in oral and dental health behavior encouraged parents to arrange preventive dental visits for their children [47].

In the present study, perceived behavioral control was defined as the students’ ability to display brushing behavior in situations, such as on trips, during an illness, in the state of boredom, when attending school, or when not having a toothpaste. The intervention program was shown to be effective in promoting their ability. Makvand et al. developed an effective oral and dental health interventional program to enhance perceived behavioral control in mothers of children aged 1–2 [48].

In the present study, the mean score of the brushing skill was 47.2% before the intervention. The percentage of this construct was 89.3% after the intervention, which was significantly higher than that before the intervention. In the study of Levin et al. the intervention program significantly increased the brushing skill from 26.1 to 87.7% [49].

In the present study, the students’ behavioral intention scores increased significantly after the educational intervention. In the study by Ebrahimipour et al. oral and dental health education based on the theory of planned behavior increased the behavioral intention score of pregnant women from 17.3 ± 2.5 to 21.4 ± 2 [50]. Behavioral preferences were the major effective variables in displaying brushing behavior, so it is suggested that parents consider children’s behavioral preferences. According to the perspective of the experts in the qualitative research, the behavioral preferences of children, such as brushing outside the toilet, having a colorful and attractive toothbrush, being able to choose toothbrushes and toothpastes, and brushing with parents must be taken into consideration.

### Reinforcing factors

The most important reinforcing factors identified in this study were social support and social reinforcement. By holding training classes for parents, teachers, and school educators, we sought to guide, assist, remind, and encourage the students to brush their teeth on a regular basis.

## Conclusions

Based on phases one to three of the model, the major predisposing factors identified in this study were knowledge, attitude, perceived susceptibility, perceived severity, perceived benefits, subjective norms, observational learning, and motivation to comply. Constructs, such as perceived barriers, proper brushing skills, perceived behavioral control, and behavioral preferences were the enabling factors. In addition, the constructs of social reinforcement and social support were the reinforcing factors based on the findings of the qualitative part of the study.

In phases four and five, the educational program was designed and implemented. According to the sixth and seventh phases, process and impact were performed. The results of the intervention indicate that, intervention program based on the community-based participatory PRECEDE-PROCEED planning model was effective in promoting tooth-brushing behavior, as well as predisposing, enabling, and reinforcing factors. Therefore, considering the importance of planning and its role in the effectiveness of intervention programs, it is suggested that participatory planning patterns such as, the PRECEDE-PROCEED model, be used in designing intervention programs.

## Supplementary Information

**Additional file 1.** Questionnaire.

## Data Availability

The datasets generated and analyzed during the current study are available from the corresponding author on a reasonable request through an E-mail (mnasirzadeh13@yahoo.com).

## Abbreviations

PRECEDE: Predisposing, reinforcing and enabling constructs in educational diagnosis and evaluation
PROCEED: Policy, regulatory, and organizational constructs in educational and environmental development
