# Supplementary material for: Design, implementation, and evaluation of a PRECEDE-PROCEED model-based intervention for oral and dental health among primary school students of Rafsanjan city: a mixed method study
Source: BMC Public Health. 2021 Sep 3;21:1609. doi: 10.1186/s12889-021-11585-z (PMC8414745; doi:10.1186/s12889-021-11585-z)
Supplement: Supplementary file 1 — Additional file 1. Questionnaire. [file 12889_2021_11585_MOESM1_ESM.docx]

**Questionnaire**

**Designing, Implementation and Evaluation of PRECEDE PROCEED Model-based Intervention on Oral and Dental Health among Primary Schools’ Students of Rafsanjan City**

**Knowledge**

**What is the function of teeth?**

1- Chewing food Yes, no, I do not know

2- Speech Yes, no, I do not know

3- Maintain facial beauty Yes, no, I do not know

**What are the effective factors in causing caries?**

4- Sugary substances Yes, no, I do not know

5- Microbes Yes, no, I do not know

6- The strength or material of the teeth Yes, no, I do not know

7- How long the food stays in the teeth Yes, no, I do not know

**How can we tell if our teeth are decaying?**

8- The presence of brown and black spots on the teeth and perforation of the teeth Yes, no, I do not know

9- Sensitivity of teeth to heat and cold Yes, no, I do not know

10- Bleeding from the gums when brushing or toothache Yes, no, I do not know

**What is the complication of tooth decay?**

11- Psychological problems such as embarrassment Yes, no, I do not know

12- Physical problems such as halitosis Yes, no, I do not know

**13- When should oral hygiene behavior begin?**

A: Before the teeth grow

B: From 6 months at the same time as the first tooth in the mouth

A: Since all the teeth came in

D: I do not know

**14- What is the most important way to remove teeth plaque?**

A: Brushing regularly and correctly

B: Washing only with water

C: remove Plaque by a dentist

D: I do not know

**15- What is the minimum time for your brushing?**

A: 2 minutes

B: 3 minutes

C: 4 minutes

D: I do not know

**16- When brushing, how much toothpaste do we need to put on the toothbrush?**

A: the size of a pea

B: the size of a half toothbrush

C: As long as the toothbrush

D: I do not know

**17- How often should we change our toothbrush?**

A: Every 2-3 months

B: Every 3-4 months

C: Every 6 months

D: I do not know

**18- How should we brush the** External surfaces of upper teeth?

A: By moving the toothbrush back and forth

B: From top to bottom

C: From bottom to top

D: I do not know

**19- How many times a day should we brush our teeth?**

A: Once

B: Twice

C: Three times and more

D: I do not know

**20- When is it better to brush?**

A: Only after eating lunch

B: Only after eating dinner

C: After eating breakfast, lunch and dinner and eating sweets and snacks

D: I do not know

**21- How often should children use fluoridated mouthwash?**

A: Once a week

B: Once a month

C: Twice a week

D: I do not know

**22- To examine their teeth, how often should refer to the dentist?**

A: Once every three months

B: Once every 6 months

C: Whenever a tooth is damaged

D: I do not know

**23- What is the best way to clean between teeth?**

A: Brushing well

B: Using dental floss

C: Using mouthwash

D: I do not know

**24- How many times a day should we floss, at least?**

A: Once

B: Twice

C: Three

D: I do not know

**Attitude** A: I agree B: I have no opinion C: I disagree

1- I think brushing is good.

2- I feel good after brushing.

3- Brushing is very useful for dental health.

4- There is no special method of brushing, it is good to brush anyway.

5- Brushing once a day is enough.

6- After consuming sugars and sweets, it is necessary to brush immediately.

7- Toothpaste has no effect on oral health.

**Perceived susceptibility** A: I agree B: I have no opinion C: I disagree

1- If I do not brush my teeth, my teeth may decay.

2- The resistance of my teeth is good and they never decay.

3- My teeth stay healthy until old age.

**Perceived severity** A: I agree B: I have no opinion C: I disagree

1- Tooth decay is a serious disease.

2- If my teeth rot, I will have severe toothache.

3- If my teeth rot, my mouth smells bad.

4- If my teeth rot, I will look ugly.

5. If my teeth rot, I cannot speak well.

6- If my teeth rot, I may lose my friends.

7- If my teeth rot, my family will be upset with me.

8- If my teeth rot, the school health teacher will be very upset with me.

9- If my teeth rot, my homework and daily tasks will be delayed.

10- If my teeth rot, my family will have to spend a lot of money on me.

**Perceived benefits** A: I agree B: I have no opinion C: I disagree

1- Brushing prevents tooth decay.

2- Brushing causes vitality and freshness.

3- Brushing increases my self-confidence.

4- Brushing makes me have beautiful teeth.

5- Brushing makes my teeth decay later.

6- Brushing makes my teeth whiter.

7- Brushing makes my mouth fragrant.

8- Brushing makes my family love me more.

9- Brushing makes me make more friends.

10- Brushing makes my teacher like me more.

11- Brushing makes my school health instructor encourage me.

**Perceived barriers** A: I agree B: I have no opinion C: I disagree

1- I do not have enough time to brush my teeth after eating breakfast.

2- I do not have mood to brush my teeth after eating dinner.

3- I do not brush because I do not have a toothbrush and toothpaste.

4- I do not brush because I do not know the correct way to brush.

5- Brushing give me nausea, that's why I don't brush.

6- When I brush my teeth, blood comes out of my gums, that's why I don't brush.

**Subjective norms** A: I agree B: I have no opinion C: I disagree

1- My family believes that I should brush my teeth regularly.

2- According to my friends, we should brush regularly.

3- The school health teacher believes that we should brush our teeth regularly.

**Motivation to comply** A: I agree B: I have no opinion C: I disagree

1- Family opinion is important for me to brush my teeth.

2- The opinion of my friends is important for me to brush my teeth.

3- The opinion of my school health teacher is important for me to brush my teeth.

**Perceived self-efficacy** A: Yes B: So-So C: A lot

1- I can brush my teeth after breakfast.

2- I can brush my teeth after eating lunch.

3- I can brush my teeth after dinner.

4- I can brush my teeth after eating sweets.

5. I can brush properly and in the right way

**Perceived behavioral control** A: Yes B: So-So C: A lot

1- I can even if I do not have mood and patient, my toothbrush.

2- I can brush my teeth regularly while traveling.

3- I can brush regularly when I am sick.

4- If I run out of toothpaste, I can still brush regularly.

5- Even if I do not like the taste of my toothpaste, I can still brush regularly.

6- I can also brush my teeth at parties

**Observational learning** A: I agree B: I have no opinion C: I disagree

1- Because I see my family brushing their teeth, I also brush my teeth.

2- Because I see my friends brushing their teeth, I also brush.

3- Because I see children brushing their teeth in cartoons, I also brush my teeth

**Social support** A: I agree B: I have no opinion C: I disagree

1- My parents guide me while brushing my teeth.

2- My parents supervise my brushing.

3- My parents help me with brushing.

4- My parents remind me when to brush my teeth.

**Social reinforcement** A: I agree B: I have no opinion C: I disagree

1- My parents always encourage me to brush my teeth.

2- My teacher encourages me to brush my teeth.

3- The school health instructor encourages me to brush my teeth.

4- My friends encourage me to brush my teeth

**Intention to behave** A: Yes B: No

1- I decide to brush three times a day.

2. I decide to brush my teeth after breakfast.

3- I decide to brush my teeth after eating lunch.

4- I decide to brush my teeth before going to bed.

**Behavioral preferences** A: I agree B: I have no opinion C: I disagree

1- I like to brush my teeth in the bathroom.

2- I like to brush my teeth in the kitchen.

3- If my toothbrush is colorful and attractive, I would rather brush my teeth.

4- If I choose and buy my own toothbrush and toothpaste, I would rather brush my teeth.

5- I like to brush my teeth with my parents.

**Checklist for controlling the correct brushing skills- Behavioral skills**

| **Row** | **Skills components** | **Yes** | **No** |
| --- | --- | --- | --- |
| 1 | He washes his toothbrush and puts toothpaste the size of a pea on it. |  |  |
| 2 | Place the toothbrush at a 45-degree angle on the outer surfaces of the upper teeth and start brushing up and down from the last tooth. |  |  |
| 3 | Place the toothbrush at a 45 degree angle on the inner surfaces of the upper teeth and start brushing from the last tooth. |  |  |
| 4 | The masticatory surfaces brush the upper teeth in a longitudinal (reciprocating) motion. |  |  |
| 5 | Place the toothbrush at a 45-degree angle on the outer surfaces of the lower teeth and start brushing from the last tooth by moving it up and down. |  |  |
| 6 | Place the toothbrush at a 45-degree angle on the inner surfaces of the lower teeth and start brushing from the last tooth by moving it up and down. |  |  |
| 7 | The masticatory surfaces brush the lower teeth in a longitudinal (reciprocating) motion. |  |  |
| 8 | Brush each tooth at least 3 times using the above methods. |  |  |
| 9 | He brushes his tongue by moving backwards in front of the toothbrush |  |  |
| 10 | Observe the duration of brushing for 3 to 5 minutes. |  |  |
| 11 | You clean your toothbrush. |  |  |
| 12 | You clean your mouth. |  |  |

**Checklist - Evaluation of brushing behavior in the last week- Behavior**

|  | Friday | Thursday | Wednesday | Tuesday | Monday | Sunday | Saturday |
| --- | --- | --- | --- | --- | --- | --- | --- |
| Morning | 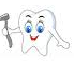 | 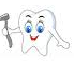 | 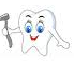 | 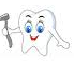 | 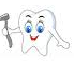 | 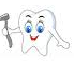 | 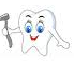 |
| Noon | 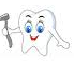 | 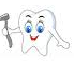 | 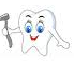 | 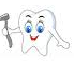 | 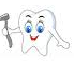 | 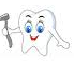 | 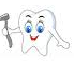 |
| the night | 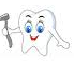 | 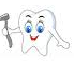 | 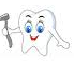 | 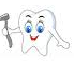 | 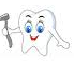 | 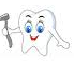 | 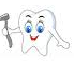 |
